# Supplementary material for: Understanding the pathways to text generation: A longitudinal study on executive functions, oral language, and transcription skills from kindergarten to first grade
Source: PLoS One. 2024 Dec 27;19(12):e0315748. doi: 10.1371/journal.pone.0315748 (PMC12140084; doi:10.1371/journal.pone.0315748)
Supplement: S1 Table — (DOCX) [file pone.0315748.s001.docx]

**Table 1. Unstandardized and standardized path coefficients for transcription skills, narrative competence, executive functions, and productivity.**

| Path | Unstandardized | Standardized |
| --- | --- | --- |
| Narrative Competence |  |  |
| Narrative Competence→ Productivity | 0.023 | 0.009 |
| Narrative structure | 1.000^+^ | 0.514^***^ |
| Unique words | 7.229 | 0.957^***^ |
| T-Units | 1.517 | 0.913^***^ |
| Total number of Words | 14.439 | 0.998^***^ |
| Transcription skills |  |  |
| Transcription skills→ Productivity | 0.163 | 0.381^***^ |
| Phoneme Isolation | 1.000^+^ | 0.783^***^ |
| Letter copying | 0.071 | 0.395^***^ |
| Name writing | 0.141 | 0.574^***^ |
| Picture word writing | 1.007 | 0.613^***^ |
| Phoneme segmentation | 0.655 | 0.598^***^ |
| Executive function |  |  |
| Executive function → Productivity | 0.343 | 0.271^*^ |
| Attention | 1.000^+^ | 0.533^***^ |
| Digit spam backward | 0.401 | 0.632^***^ |
| Oral cloze task | 0.142 | 0.438^***^ |
| Inhibitory control | 0.036 | 0.559^***^ |
| Cognitive flexibility | 0.004 | 0.199^*^ |
| Productivity |  |  |
| Unique words written | 1.000^+^ | 0.953^***^ |
| Correctly written sequences | 1.351 | 0.957^***^ |
| Written Word fluency | 0.367 | 0.904^***^ |
| Total number of written words | 1.975 | 0.699^***^ |

^+^Fixed parameter; **p* < .05; ***p* < .01; ****p* < .001
