# Supplementary material for: Understanding the pathways to text generation: A longitudinal study on executive functions, oral language, and transcription skills from kindergarten to first grade
Source: PLoS One. 2024 Dec 27;19(12):e0315748. doi: 10.1371/journal.pone.0315748 (PMC12140084; doi:10.1371/journal.pone.0315748)
Supplement: S2 Table — (DOCX) [file pone.0315748.s002.docx]

**Table 2. Unstandardized and standardized path coefficients for transcription skills, narrative competence, executive functions, and quality**

| Path | Unstandardized | Standardized |
| --- | --- | --- |
| Narrative Competence | | |
| Narrative Competence→ Quality | 0.285 | 0.184^*^ |
| Narrative structure | 1.000^+^ | 0.515^***^ |
| Unique words | 7.182 | 0.956^***^ |
| T-Units | 1.494 | 0.915^***^ |
| Total number of words | 14.373 | 0.998^***^ |
| Transcription skills | | |
| Transcription skills→ Quality | 0.091 | 0.344^*^ |
| Phoneme isolation | 1.000^+^ | 0.777^***^ |
| Letter copying | 0.071 | 0.427^***^ |
| Name writing | 0.141 | 0.565^***^ |
| Picture word writing | 1.007 | 0.593^***^ |
| Phoneme segmentation | 0.655 | 0.611^***^ |
| Executive function | | |
| Executive function → Quality | 0.229 | 0.291 |
| Attention | 1.000^+^ | 0.521^***^ |
| Digit spam backward | 0.401 | 0.605^***^ |
| Oral cloze task | 0.142 | 0.430^***^ |
| Inhibitory control | 0.036 | 0.575^***^ |
| Cognitive flexibility | 0.004 | 0.205^*^ |
| Quality |  |  |
| Written narrative structure | 1.000^+^ | 0.791^***^ |
| Written sentence fluency | 0.052 | 0.165 |
| Percentage of correctly written words | 2.192 | 0.346^***^ |
| Number of written casual connectors | 1.975 | 0.449^***^ |

^+^Fixed parameter; **p* < .05; ***p* < .01; ****p* < .001
