# Supplementary material for: Understanding the pathways to text generation: A longitudinal study on executive functions, oral language, and transcription skills from kindergarten to first grade
Source: PLoS One. 2024 Dec 27;19(12):e0315748. doi: 10.1371/journal.pone.0315748 (PMC12140084; doi:10.1371/journal.pone.0315748)
Supplement: S3 Table — (DOCX) [file pone.0315748.s003.docx]

**Table 3. Unstandardized and standardized path coefficients for transcription skills, narrative competence, executive functions, and syntactic complexity.**

| Path | Unstandardized | Standardized |
| --- | --- | --- |
| Narrative Competence |  |  |
| Narrative Competence→ Syntactic Complexity | 0.070 | 0.191^*^ |
| Narrative structure | 1.000^+^ | 0.515^***^ |
| Unique words | 7.294 | 0.957^***^ |
| T-Units | 1.532 | 0.912^***^ |
| Total number of Words | 14.612 | 0.999^***^ |
| Transcription skills |  |  |
| Transcription skills→ Syntactic Complexity | 0.012 | 0.202 |
| Phoneme Isolation | 1.000^+^ | 0.787^***^ |
| Letter copying | 0.067 | 0.378^***^ |
| Name writing | 0.131 | 0.539^***^ |
| Picture word writing | 0.991 | 0.605^***^ |
| Phoneme segmentation | 0.667 | 0.607^***^ |
| Executive function |  |  |
| Executive function → Syntactic Complexity | 0.080 | 0.460^**^ |
| Attention | 1.000^+^ | 0.548^***^ |
| Digit spam backward | 0.356 | 0.583^***^ |
| Oral cloze task | 0.143 | 0.456^***^ |
| Inhibitory control | 0.036 | 0.560^***^ |
| Cognitive flexibility | 0.004 | 0.203^*^ |
| Syntactic complexity |  |  |
| Grammatical structure | 1.000^+^ | 0.712^***^ |
| Written T-units | 3.615 | 0.641^***^ |

^+^Fixed parameter; **p* < .05; ***p* < .01; ****p* < .001
