# Supplementary material for: Understanding the pathways to text generation: A longitudinal study on executive functions, oral language, and transcription skills from kindergarten to first grade
Source: PLoS One. 2024 Dec 27;19(12):e0315748. doi: 10.1371/journal.pone.0315748 (PMC12140084; doi:10.1371/journal.pone.0315748)
Supplement: Attachment — Submitted filename: Response to Reviewers.docx [file pone.0315748.s006.docx]

Dear Dr. Tsang,

We would like to thank you for the letter dated septembre 20, 2024, and the opportunity to resubmit a revised manuscript. With this cover letter, we are submitting the revised manuscript PONE-D-24-27767 “Understanding the pathways to text generation: A longitudinal study on executive functions, oral language, and transcription skills from kindergarten to first grade”, for publication in the journal PLOS ONE. We have addressed the suggestions from both the editor and the reviewers to the best of our abilities, and have revised the manuscript accordingly.

Sincerely,

The first author, also on behalf of the co-authors

Reviewers' comments:

Reviewer #1: In general, it is a good manuscript, with important practical implications. As a consideration, I would like to know more information abouta participants. I consider that some sociodemographic characteristisc should be considered. In addition, it could be interesting to expand the study to other regions of Spain, in order to obtain more robust conclusions.

R= Thank you for your positive evaluation of our manuscript and for your thoughtful suggestions. Regarding your request for additional information about the participants, we would like to clarify that in the "Participants" section of the manuscript, we have already provided relevant sociodemographic details such as age, gender, socioeconomic status, and the educational background of the parents. However, we acknowledge that ethnicity is not a relevant factor in the context of our study, which is why it was not included. Additionally, we have now specified that all participants were native Spanish speakers to provide further clarity.

We appreciate your suggestion to expand the study to other regions of Spain, and we have recognized this in the "Limitations" section, as well as under "Future Research Directions," where we address the need to extend the research to other regions to strengthen the robustness of our findings.

Thank you once again for your valuable feedback.

Reviewer #2:

Thanks for inviting me to review this paper “Understanding the pathways to text generation: A longitudinal study on executive functions, oral language, and transcription skills from kindergarten to first grade”. Overall, it presented an interesting exploration for extending the Not-so-simple view of writing. Although the author has provided some interesting insight for explaining their finding, I still found that there are much to address and would not be able to recommend this study to be published in the journal “PLoS ONE” at this moment.

1. “Introduction”. Please elaborate on why writing production is a multidimensional phenomenon and how it is connected with the focus of dimensions other than writing quality.

R= Thank you for your thoughtful feedback. In response to your suggestion, we have elaborated on why writing production is considered a multidimensional phenomenon and how it is connected to dimensions beyond writing quality. Specifically, we have added a paragraph in the introduction that outlines the three key dimensions of writing development: productivity, quality, and syntactic complexity. We also cite studies such as Kim et al. (2014), Wagner et al. (2011), and Olinghouse and Graham (2009) to support this multidimensional view, emphasizing the interplay between these dimensions in early childhood writing development. This addition highlights the importance of analyzing writing from a broader perspective to fully understand children’s writing development. We hope this addresses your concern.

2. “Introduction”. Please explain the necessity of extending Kim et al.’s study. How does research of this type contribute to the field? In the other sections of the manuscript, the authors claim to extend the Not so simple view of writing.

R= In the revised introduction, we have highlighted the multidimensional nature of writing production, emphasizing that it encompasses productivity, quality, and syntactic complexity. While Kim et al. (2014) provided valuable insights into these dimensions within an English-speaking context, we noted a gap in understanding how these dimensions manifest in other linguistic environments, particularly in Spanish, which features a shallow orthography. By extending this research to Spanish-speaking students, our study addresses this gap and explores whether the patterns identified by Kim et al. hold true across different linguistic contexts. Furthermore, we have articulated how our investigation into the interplay between transcription skills, oral language abilities, and executive functions contributes to a more comprehensive understanding of early writing development. This aligns with the 'Not So Simple View of Writing,' reinforcing the complexity of cognitive, linguistic, and motor skills involved in writing production.

We believe these additions effectively clarify the necessity of our study and its contribution to the field. Thank you once again for your valuable comments, which have helped us strengthen our manuscript.

3. “Literature review”. The author has included a substantial amount of evidence in the literature review, giving it the potential to become a solid and informative review. However, I am not very convinced on some arguments such as “ few studies have explored the significance of executive functions” given the substantial studies (even including meta-analysis studies) have done. Meanwhile, since I am not an expert in Spanish, may I know if there is any language that is with shallow orthography, where relevant studies on similar topics have been done?

R=Thank you for your valuable feedback regarding our discussion on the significance of executive functions in writing development. We appreciate your point about the substantial existing research in this area. In response, we have revised the manuscript to clarify our argument. While acknowledging that several studies have investigated executive functions, we emphasize the need for further exploration of their specific roles in writing development, especially since more extensive research has been conducted on transcription and oral language skills. We believe this nuanced approach strengthens our literature review and better reflects the current state of research.

4. “Methodology”. Please include more detail related to the measurement of syntactic complexity. Instead of common calculations such as TTR, the study assigns weight to different types of sentences. What is the specific rationale for assigning such a weight to the sentence? In addition, please provide an example of each class of sentence and explain why the sentence was identified in a particular way.

R= Thank you for your insightful feedback regarding the measurement of syntactic complexity. In response to your request, we have included additional details in the Methods section of the manuscript. Specifically, we clarified the rationale for assigning different weights to sentence types and provided examples of each type—simple, compound, complex, and compound-complex sentences—to illustrate how the scoring reflects increasing linguistic complexity in children's writing. The updated description also explains how our approach, based on Granowsky and Botel (1974) but modified to include compound-complex sentences, allows for a more nuanced evaluation of syntactic sophistication in young students. We trust that this revision addresses your concern and enhances the clarity of our scoring methodology.

5. “Methodology” The authors provided reliability for some tasks. Please provide reliability values for remaining tasks.

R= we added the reliability coefficients for the remaining tasks such as narrative text composition, oral cloze task, and cognitive flexibility

6. “Results”. The authors claimed that “all absolute values of skewness and kurtosis remained below 3.0 and 10.0”, however, it is not the truth in Table 1.

R= Thank you for your valuable feedback regarding our claim about the distribution of variables in Table 1. We acknowledge that some variables exceeded the recommended thresholds for skewness and kurtosis as indicated. To address this issue, we have revised the manuscript to clarify that, despite these violations of normality, we employed the Robust Maximum Likelihood (MLR) estimation method to account for the non-normality of the data. This approach adjusts standard errors and fit indices, which is a widely accepted practice in the structural equation modeling literature. We appreciate your insight, as it has helped us improve the clarity and rigor of our results section.

7. “Results”. Please include a measurement model before presenting the three models.

R= We have integrated the requested information into the Results section, including a measurement model before presenting the three analytical models. In this revised section, we describe the exogenous variables of the measurement model: executive functions, transcription skills, and oral narrative competence. We have also incorporated our hypotheses directly into the description of these variables, highlighting our expectation that executive functions and oral narrative competence have a greater influence on writing outcomes than transcription skills, given the context of the Spanish orthography. Additionally, we have provided a comprehensive diagram illustrating the measurement model, which shows the relationships among latent variables, their observable indicators, and the interconnections between endogenous and exogenous constructs. We believe these modifications enhance the clarity and cohesion of the section and hope they meet your expectations.

8.” Results”. There are some notable issues in the models, for instance, the surprising low loadings for cognitive flexibility across EF latent variables.

R= In the revised manuscript, we have added a section that explores potential explanations for this low loading. We discuss how cognitive flexibility may develop at a slower rate than other executive functions such as inhibitory control and working memory in early childhood. Additionally, we considered the possibility that the tasks used to measure cognitive flexibility may not adequately capture age-appropriate manifestations of this ability. Finally, we acknowledge that, while executive functions are often viewed as a unitary construct in early childhood, cognitive flexibility may begin to differentiate slightly from other components, indicating emerging distinctions in their development. We believe that these additions strengthen our discussion and provide a more comprehensive understanding of the role of cognitive flexibility within the framework of executive functions in young children.

In addition, I am rather confused in reading the Tables and Figures. For instance, the WTU looks like insignificant in the SC model (dashed line with two digits of decimals, .64), however, the table indicated it was significant (.641, three digits of decimals). I assume the information provided in tables and figures are the same, why is it necessary to present in two different ways?

R= We understand your concerns about the discrepancies in the reporting of values, particularly regarding the WTU in the SC model. In response to your comments, we have standardized the presentation of values across all tables and figures by reducing the reported values to two decimal places. This change ensures consistency between the tables and figures, eliminating any confusion and aligning the information presented. Additionally, we have also standardized the presentation of all values to two decimal places throughout the text. We appreciate your attention to detail, which has helped us improve the clarity of our results. We hope that these adjustments address your concerns.

9. “Discussion and implications”. This study only examined the relationship of writing components (Time point1) and writing performance (Time point2). I doubt that such a design could be claimed as “longitudinal”.

R= We appreciate your valuable feedback regarding the study's design. While we did not remeasure the transcription skills, oral language abilities, and executive functions in first grade, we would like to clarify that our study maintains a longitudinal framework. The focus was on examining how these initial skills measured in kindergarten (Time 1) contribute to writing performance in first grade (Time 2). This design allows us to investigate the predictive relationships between early skills and later writing dimensions—productivity, quality, and syntactic complexity—without necessitating a reassessment of the predictors. By analyzing the impact of these kindergarten abilities on later writing outcomes, we aim to provide insights into the developmental progression of writing skills. We believe this approach is consistent with longitudinal research, as it explores the influence of earlier skills on subsequent performance over time.

10. “Discussion and implications”. More studies could be linked in the discussion section.

For instance, you can compare your findings with previous studies in discussion between lines 771 to 783.

R= We appreciate your suggestion to include more studies in the discussion section and compare our findings with previous research, particularly between lines 771 and 783. We have incorporated a broader discussion of relevant studies, such as Oddsdottir et al. (2021), Drijbooms et al. (2015), and Cordeiro et al. (2020), which explore the relationship between executive functions and writing across different ages and orthographies.

Additionally, we have included a comparative analysis highlighting the differences between studies conducted in opaque orthographies, like English, and our study, which was conducted in a transparent orthography (Spanish), similar to Oddsdottir et al. (2021) in Icelandic. We argue that the early automatization of transcription skills in transparent orthographies might allow executive functions to play a more prominent role in explaining syntactic complexity in early stages of writing development. This comparison supports our hypothesis that, in transparent orthographies, syntactic complexity may be more heavily influenced by executive functioning than by transcription skills, which tend to become automatized earlier.

With these revisions, we hope to have addressed your comment appropriately and provided a more robust discussion within the context of existing literature.

Also, the explanation between lines 830 to 835 would be more convincing if the author could provide some reference.

R= Thank you for your observation regarding the need for a reference to support the explanation between lines 830 and 835. We have reviewed this section and added a reference to support the statement. Specifically, we have incorporated the study by Yeung et al. (2017), which highlights how oral narrative skills significantly predict writing performance, reinforcing the idea that both oral and written narratives share a common foundation of linguistic skills such as vocabulary, grammar, and narrative structure.

This comment is also applicable to the explanation between lines 820 to 846.

R= Thank you for your suggestion regarding the need for a reference to support the statement on the influence of executive functions (EFs) on productivity in children's writing. We have reviewed the manuscript and added an appropriate reference to substantiate this claim. Specifically, we have drawn upon the study by Zahra et al. (2023), which demonstrates how executive functions, including planning, inhibitory control, and updating, significantly contribute to narrative text production in students, particularly in terms of text length, syntactic complexity, and content quality. This reference reinforces the importance of considering cognitive processes beyond transcription skills and oral language abilities in writing development. We hope this addition adequately addresses your suggestion.

R= Thank you for your suggestion regarding the need for references to support the statement about the role of executive functions (EFs) in managing cognitive processes involved in constructing complex sentences and maintaining grammatical accuracy. We have reviewed the manuscript and added a relevant reference to substantiate this claim. Specifically, we have incorporated the study by Drijbooms et al. (2017), which highlights the significance of EFs such as working memory, cognitive flexibility, and inhibitory control in writing development. The findings of this study demonstrate that inhibition and planning are critical for managing the complexities of writing, thus reinforcing the notion that EFs are essential for constructing complex sentences and ensuring grammatical accuracy.

R= Specifically, we have incorporated findings from Drijbooms et al. (2017), which demonstrate that EFs, particularly inhibition and planning, contribute directly to the syntactic sophistication of written narratives by enabling children to manage the complexities of sentence construction. In addition, we referenced Yeung et al. (2017), which highlights how narrative competence enhances the coherence and richness of narrative content. This distinction underscores the complementary roles of EFs and narrative competence in developing high-quality written narratives.

We hope these additions adequately address your request for clarification and supporting references.

11. “Limitations”. This study did not measure students’ non-verbal intelligence (which usually did in EF related studies) nor other linguistic skills (orthographic knowledge/morphological awareness which may relate to the languages you claimed “shallow orthography”). Such limitation should be acknowledged and justified.

R= In response to your suggestion, we have added a detailed acknowledgment of the limitations related to the absence of measurements for students’ non-verbal intelligence and other linguistic skills such as orthographic knowledge and morphological awareness. We recognize that these factors are particularly relevant in the context of a shallow orthography and could significantly influence the development of writing abilities. We have emphasized the importance of incorporating these variables in future research to provide a more comprehensive understanding of the cognitive and linguistic factors affecting writing performance.
